# Multidimensional study of cell division cycle-associated proteins with prognostic value in gastric carcinoma

**DOI:** 10.17305/bjbms.2021.5783

**Published:** 2021-05-12

**Authors:** Peixin Lu, Wen Cheng, Kexin Fang, Bin Yu

**Affiliations:** 1 Department of Anesthesiology, Tongji Hospital Affiliated to Tongji University School of Medicine, Shanghai, China; 2 School of Medicine, Tongji University, Shanghai, China

**Keywords:** Gastric cancer, cell division cycle-associated protein family, bioinformatics analysis, biomarker, prognosis

## Abstract

Gastric cancer (GC) represents a widespread malignancy with a poor prognosis. Hence, discovering reliable biomarkers is necessary. The cell division cycle-associated protein (CDCA) family, comprising CDCA1–8, plays a key role in tumor progression. However, whether CDCA expression has prognostic value in GC, especially stomach adenocarcinoma (STAD), has not been elucidated yet. Consequently, we conducted a multifaceted study using bioinformatic tools aimed at exploring CDCA expression levels and appraising their potential prognostic values in patients with STAD. All eight CDCAs were significantly upregulated in STAD tissues compared with healthy tissues. Elevated CDCA4/7/8 mRNA expression predicted a short overall survival, and increased CDCA7 transcriptional levels predicted a short disease-free survival. The most frequent alteration in patients with STAD was low mRNA expression. The functional enrichment analysis incorporating the gene ontology (GO) and Kyoto encyclopedia of genes and genomes (KEGG) pathways showed that the cell cycle, foxO signaling pathway, and Epstein–Barr virus were relevant to the main functions of CDCAs. Finally, the immune infiltration analysis revealed a significant correlation between CDCA expression and the infiltration extent of six immunocytes. Therefore, differentially expressed CDCAs may represent potential biomarkers for the prognosis of patients with STAD that can improve survival. Furthermore, this study might offer new ideas for the design and development of immunotherapeutic drugs.

## INTRODUCTION

Gastric cancer (GC) represents a widespread and malignant carcinoma with the fifth highest incidence and third highest mortality rate worldwide [1]. In 2018, over 1 million new patients and nearly 800,000 deaths occurred [1]. The number of GC cases may increase in the future because of the aging population [2]. Various molecular and histological subtypes are featured in GC and are mainly subdivided into four groups: microsatellite instability, Epstein–Barr virus, diffuse, and intestinal subtypes [3]. Since the initial detection and diagnosis of GC often occurs at an already advanced stage [4], the options for surgical treatment are often narrowed, which results in worse outcomes. Targeted therapy backed by palliative treatment and chemotherapy is envisioned to be an important complementary therapy for GC to prolong the life expectancy and increase the quality of life for advanced patients with GC [5]. Moreover, the discovery of prognostic biomarkers for the diagnosis and management of GC is urgently needed.

Cell division plays an indispensable role in health and disease. Many studies have confirmed that numerous abnormalities in cell division can trigger the growth of malignant carcinomas [6-9]. The cell division cycle-associated (CDCA) protein family is deeply involved in cell division, comprising eight members, CDCA1–8. CDCA1 (also called NUF2), a component of the Ndc80 complex, is responsible for regulating mitosis and the spindle checkpoint [10]. CDCA2 can promote cancer cell proliferation via the hypoxia inducible factor-1a pathway [11]. CDCA3 has been described to control the cell cycle by degrading the endogenous cell cycle inhibitor WEE1 G2 checkpoint kinase [12,13]. Through negative feedback-modulated activator early two factors, CDCA4 operates as an essential regulatory agent in cell proliferation [14]. CDCA5 can regulate sister chromatid cohesion and separation in cell division [15]. CDCA6 (also called CBX2) binds mitotic chromosomes, enabling the inheritance of the repressive locus during cell division [16]. CDCA7 serves as an essential transcription factor that is governed by c-Myc [17], and CDCA8 is an important regulator of mitosis [18].

The prognostic values of CDCAs are well documented in several cancers, including ovarian cancer, hepatocellular carcinoma, and endometrial carcinoma [19-21]. However, the function and prognostic utility of the CDCA family in GC, especially stomach adenocarcinoma (STAD), remain unknown and elusive. Thus, we performed a comprehensive analysis of the CDCAs in STAD and examined their potential as prognostic biomarkers, which could contribute to selecting the optimal treatment for patients and thus improve outcomes.

## MATERIALS AND METHODS

### ONCOMINE

As an efficient online database, ONCOMINE (www.oncomine.org) provides powerful, genome-wide expression analysis [22] with which CDCA expression and transcriptional extents in various carcinoma types were investigated. We set the *p-*value to 0.05, the fold-change index to 2, and gene rank as top 10% for the thresholds. Student’s *t*-test was used for statistical analysis.

### Ualcan

Ualcan serves as a multifaced and integrated web source providing analysis of different cancers (http://ualcan.path.uab.edu/) [23]. Here the degrees of CDCA expression were analyzed in the “TGCA Gene analysis” modular and the “Stomach adenocarcinoma” dataset. The differences were considered statistically significant when the *p-*value is <0.05.

### GEPIA

GEPIA, an internet-based database, provides functions including differential expression, correlation, and survival analysis based on TCGA and GTEx data (http://gepia.cancer-pku.cn/) [24]. The distinguished analysis of mRNA expression in STAD versus normal tissues, pathological stages analysis, correlative prognostic analysis, and multiple gene analysis were performed with this tool. The *p*-values were obtained with Student’s *t*-test, and the Kaplan–Meier curves were selected to present the findings of the survival analysis.

### Kaplan–Meier plotter

**FIGURE 1:**
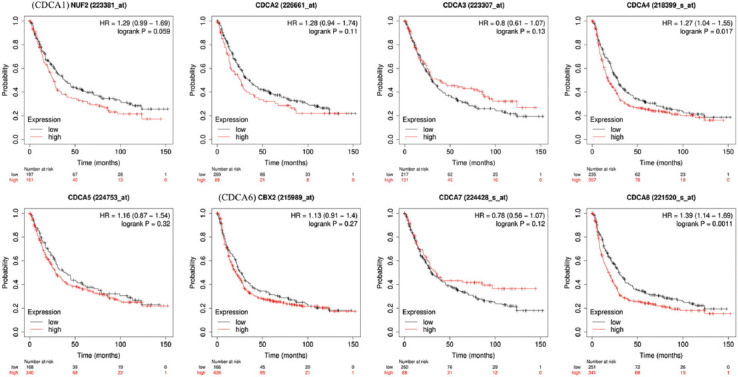
Prognostic value of cell division cycle-associated (CDCA) proteins in stomach adenocarcinoma (STAD). Increased levels of CDCA4 (*p* = 0.0017) and CDCA8 (*p* = 0.0011) were correlated with shorter overall survival of patients with STAD.

The Kaplan–Meier (KM) plotter allows users to estimate the impact of the expression of over 50,000 genes on the survival of patients with GC (http://kmplot.com/analysis/) [25]. The prognostic value of CDCAs in patients with STAD was investigated. The *p*-value, hazard ratio index, 95% confidence interval, and number of people at risk are displayed in the Figure 1. The differences were considered statistically significant when the *p-*value is <0.05.

### cBioPortal

cBioPortal is an accessible tool for browsing multidimensional cancer datasets (www.cbioportal.org) [26]. Backed by the TCGA database, the altered CDCAs in the STAD samples were derived from the cBioPortal. We also obtained the mutation type and corresponding alteration details of each protein (selecting the data with the highest mutation frequency).

### PolyPhen-2 and PROVEAN

PolyPhen-2 (http://genetics.bwh.harvard.edu/pph2/) helps users explore the influences of missense mutations on protein functions [27], and PROVEAN (http://provean.jcvi.org/index.php) provides predicted function analysis of gene mutations [28]. Using these two tools, we measured the effect of CDCA mutations on protein functions.

## STRING

STRING (https://string-db.org/) is a library of predictive associations between proteins, including physical and functional associations [29]. We conducted a protein–protein interaction (PPI) network for CDCAs and obtained 50 relative genes for further investigation.

### GeneMANIA

GeneMANIA acts as an internet-based resource designed to help users explore the possible relationships between the genes of interest (http://www.genemania.org) [30]. The predictive values of CDCAs were analyzed via GeneMANIA.

### David 6.8 and Hiplot

David 6.8 offers users a thorough set of functionality-commenting tools to elucidate the biofunctions of genes (https://david.ncifcrf.gov/) [31]. We conducted functional enrichment analysis including GO and KEGG for CDCAs and 50 relative genes. Then, the results obtained from David 6.8 were visualized using Hiplot, a scientifically based resource for information analysis (https://hiplot.com.cn/). The GO analysis comprises three parts: biological process (BP), cellular components (CC), and molecular function (MF).

## TIMER

TIMER is a user-friendly tool that provides a systematized evaluation of immunological infiltrating degrees in various carcinomas (https://cistrome.shinyapps.io/timer/) [32]. The relationships of CDCA expression levels and immunological infiltrating levels in patients with STAD were carried out in the Gene module and shown as scatterplots.

### Statistical analysis

Student’s *t*-test was used to perform the expression analysis for CDCAs in STAD with ONCOMINE, UALCAN, and GEPIA. One-way analysis of variance was used to analyze the expression levels of CDCAs in different stages of STAD. Survival analyses including overall survival (OS) and disease-free survival (DFS) were conducted with KM plots and GEPIA using log-rank tests. The infiltration association analysis was conducted using the Spearman correlation coefficient. The *p*-values of <0.05 indicated statistical significance.

### Ethical statement

No local ethical approval, official statement, or informed consent were necessary since the clinical data were obtained in a publicly available manner from the TCGA database.

## RESULTS

### Overexpression of CDCA mRNA in patients with STAD

**FIGURE 2:**
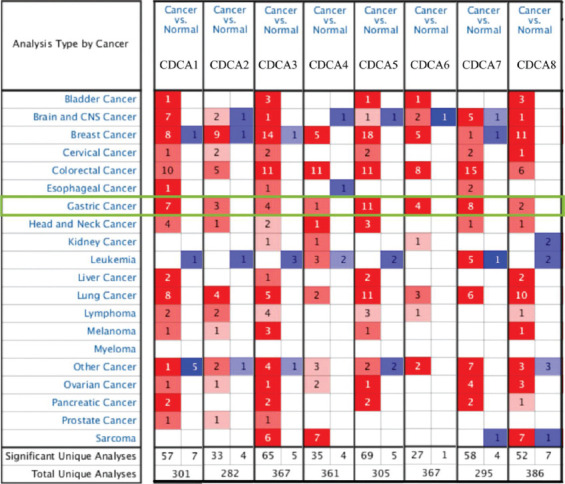
Expression profile of cell division cycle-associated (CDCA) mRNA in cancer tissues (ONCOMINE). The counts of data sets with significant changes in CDCA mRNA expression are either elevated (red) or reduced (blue). The transcript levels of CDCA1–8 were upregulated in gastric cancer tissues (green frame).

ONCOMINE was selected to analyze the distinct CDCA expression in patients with STAD (Figure 2). In comparison with paired healthy tissues, the transcriptional levels of all CDCAs were markedly elevated in STAD tissues, which is consistent with the data in Table 1. For instance, from DErrico’s dataset, CDCA1 mRNA expression appeared upregulated in diffuse gastric adenocarcinoma, gastric mixed adenocarcinoma, and gastric intestinal-type adenocarcinoma, for which the matching fold changes were 2.858, 4.498, and 5.515, respectively. Additionally, CDCA2 transcriptional levels were significantly elevated in gastric mixed adenocarcinoma and gastric intestinal-type adenocarcinoma, for which the fold changes were 3.287 and 3.851, respectively [33].

**TABLE 1:**
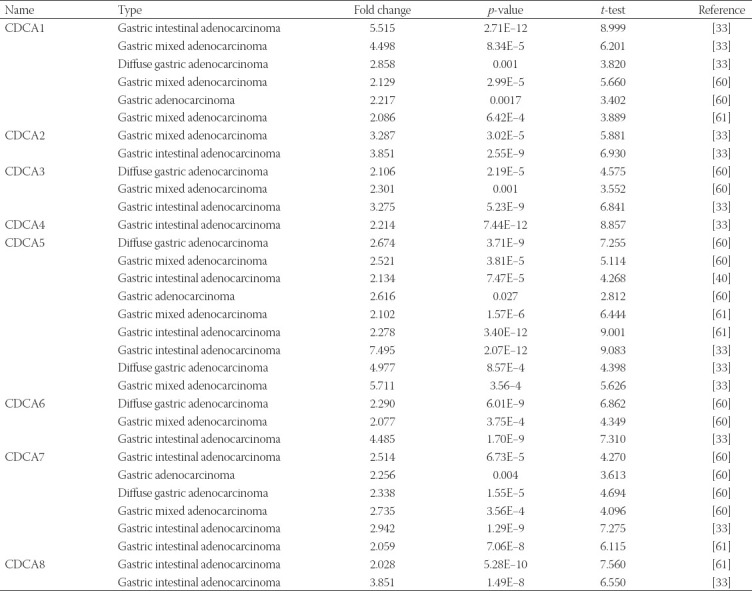
Cell division cycle-associated protein (CDCA) transcript levels in various types of stomach adenocarcinoma (ONCOMINE).

**FIGURE 3:**
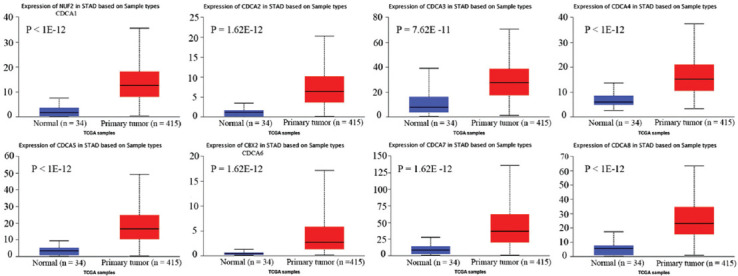
Cell division cycle-associated (CDCA) expression levels in stomach adenocarcinoma (STAD) (UALCAN). CDCA1–8 expression levels were significantly upregulated in STAD versus normal tissues (*p* < 0.05).

**FIGURE 4:**
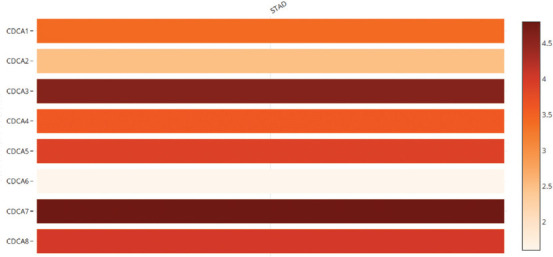
The relative levels of cell division cycle-associated (CDCA) protein-2 in stomach adenocarcinoma (GEPIA). CDCA7 was the most upregulated CDCA.

CDCA transcription levels in STAD tumors and normal tissues were also estimated with UALCAN. The findings indicated significant upregulation of the mRNA levels of CDCA1 (*p* < 1E−12), CDCA2 (*p* = 1.62E−12), CDCA3 (*p* = 7.62E−11), CDCA4 (*p* < 1E−12), CDCA5 (*p* < 1E−12), CDCA6 (*p* = 1.62E−12), CDCA7 (*p* = 1.62E−12), and CDCA8 (*p* < 1E−12) (Figure 3). CDCA7 mRNA levels were the most upregulated in comparison with the other CDCAs in STAD tissues (Figure 4).

**FIGURE 5:**
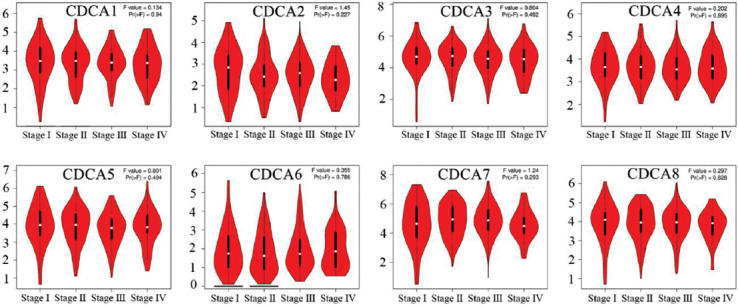
Correlations between various cell division cycle-associated (CDCA) proteins and pathological phases of stomach adenocarcinoma (STAD) (GEPIA). CDCAs did not change notably during the different phases of STAD.

Subsequently, we assessed the connections between differentially expressed CDCAs and the different pathologic phases of patients with STAD using GEPIA. However, these connections did not change significantly during the different phases of STAD (Figure 5).

### Prognostic value of CDCAs in patients with STAD

**FIGURE 6:**
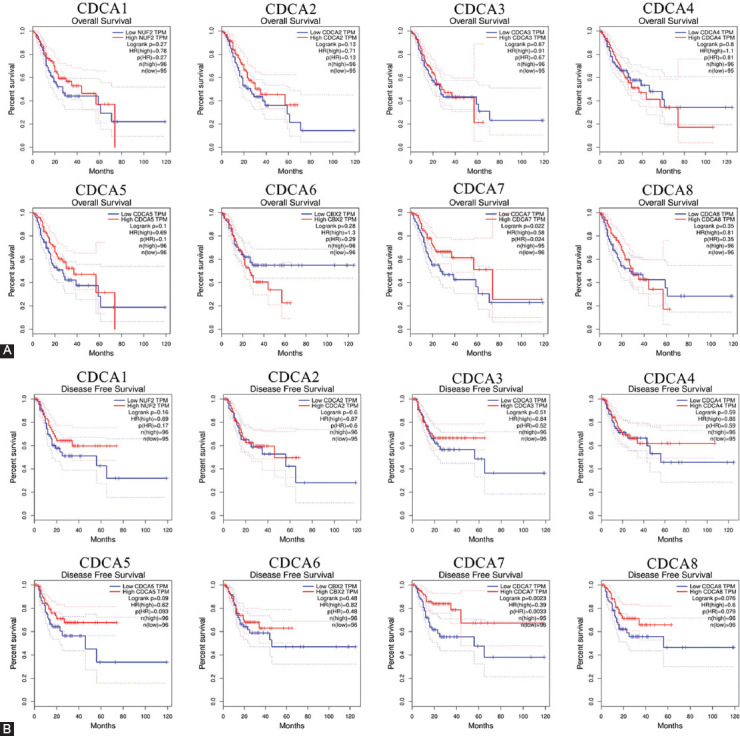
The prognostic value of cell division cycle-associated proteins (CDCAs) in stomach adenocarcinoma (STAD) (GEPIA). (A) The overall survival (OS) curves of CDCAs. Elevated CDCA7 transcript levels were linked with poorer OS (*p* = 0.022). (B) The disease-free survival (DFS) curves of CDCAs. Patients with STAD and upregulated CDCA7 levels had shorter DFS (*p* = 0.0023).

To investigate the role of CDCA expression in the development of STAD, we analyzed the correlation between CDCA and the clinical results with GEPIA. Patients with elevated CDCA7 expression had significantly shortened OS (*p* = 0.022; Figure 6A). Furthermore, patients with STAD and high CDCA7 expression had significantly shortened DFS (*p* = 0.0023; Figure 6B). Except for CDCA7, other CDCAs did not affect OS or DFS.

Next, the KM plotter was used to study the implications of CDCAs on the outcomes of patients with STAD. High transcriptional levels of CDCA4 (HR = 1.27, *p* = 0.017) and CDCA8 (HR = 1.39, *p* = 0.0011) were significantly linked to lower OS in patients with STAD (Figure 1).

### Mutations, PPI networks, and predicted protein functions of CDCAs in patients with STAD

**FIGURE 7:**
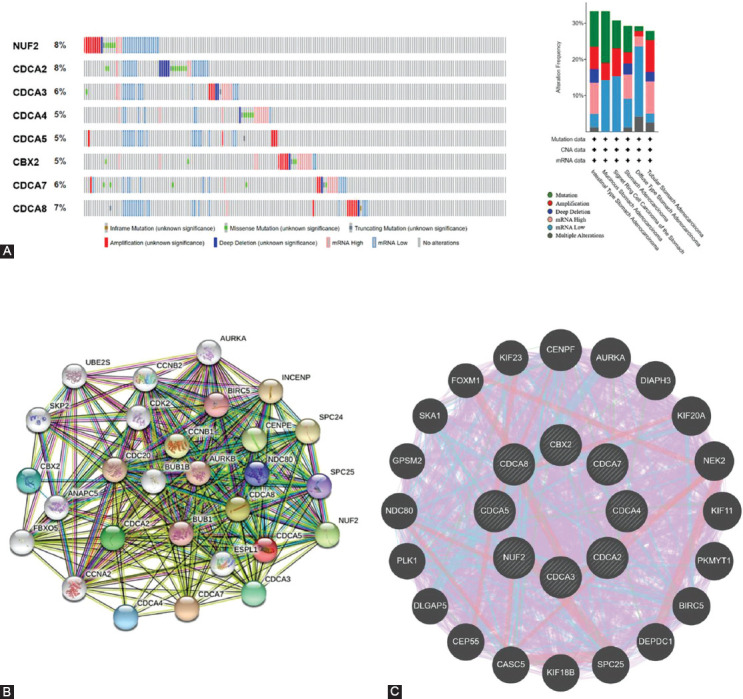
Changes in genes and protein–protein interaction (PPI) networks of cell division cycle-associated proteins (CDCAs) in stomach adenocarcinoma (STAD) (cBioPortal, STRING, and GeneMANIA). (A) Summarized changes of variously expressed CDCAs in STAD. Low mRNA was the most common change. (B, C) PPI networks of CDCAs.

**FIGURE S1:**
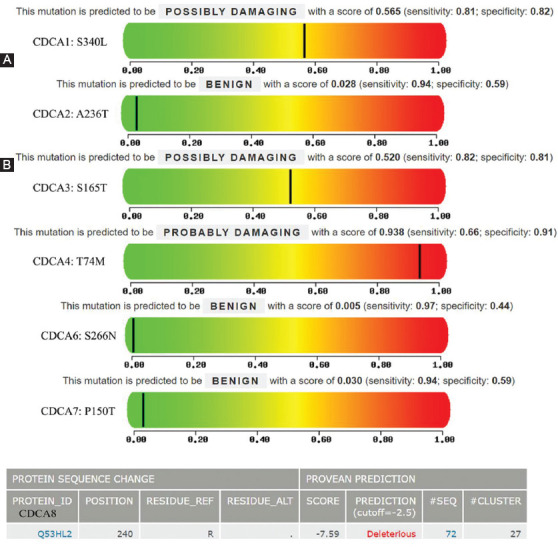
Effect of gene mutations on cell division cycle-associated (CDCA) protein functions. (A) The missense mutations of CDCA1 (score = 0.565), CDCA3 (score = 0.520), and CDCA4 (score = 0.938) showed disruptive effects on protein functions (PolyPhen-2). The higher the score is, the greater the likelihood of damage. (B) The nonsense mutation of CDCA8 was predicted to be deleterious for protein functions.

The gene variations of CDCAs in STAD cases were analyzed using cBioPortal. The respective changes for CDCA1 (NUF2), CDCA2, CDCA3, CDCA4, CDCA5, CDCA6 (CBX2), CDCA7, and CDCA8, constituted 8%, 8%, 6%, 5%, 5%, 5%, 6%, and 7% of the STAD samples, respectively (Figure 7A). The most frequent variation in the samples was mRNA downregulation (Figure 7A). Subsequently, predicted protein function analysis was performed to assess the pathogenicity of CDCA gene mutations (with PolyPhen-2 and PROVEAN). We found that the missense mutations of CDCA1 (score: 0.565) and CDCA3 (score: 0.520) were possibly damaging, whereas the missense mutation of CDCA4 (score: 0.938) was probably damaging to the protein functions (Figure S1A). The nonsense mutation of CDCA8 was predicted to be deleterious to the protein functions (Figure S1B).

Next, the underlying interconnections of different CDCAs were identified via PPI network charts using STRING (Figure 7B). The functionality of these variously expressed CDCAs was implicated in the cell cycle. Furthermore, the results of GeneMANIA demonstrated that the functions of CDCAs and their corresponding molecules (PLK1, NDC80, SKA1, KIF23, et al) were linked to mitosis, nuclear division, and organelles (Figure 7C).

### Predicted functional and pathway enrichment assessment for CDCAs in patients with STAD

**FIGURE 8:**
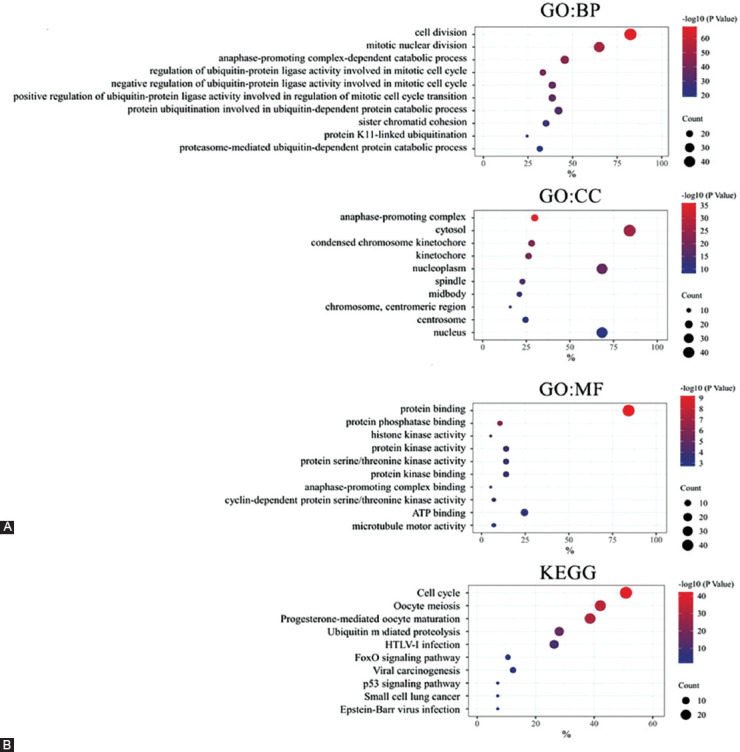
Functionally enriched analysis including GO and KEGG pathways for various cell division cycle-associated proteins (CDCAs) and 50 relative genes in stomach adenocarcinoma (STAD) (David 6.8 and Hiplot). (A) Bubble charts of the biological process (BP) group, cellular components (CC) group, and molecular function (MF) group of the GO. (B) Bubble charts of the KEGG pathway.

CDCAs and 50 related genes from STRING were analyzed using DAVID 6.8 and the Hiplot tool. The items with the highest enrichment in the BP group were cell division, mitotic cell cycle, anaphase-promoting complex-dependent catabolic process, modulation of ubiquitin protein ligase activity engaged with mitotic cell cycle, protein ubiquitination engaged with ubiquitin-dependent protein catabolic process, sister chromatid cohesion, protein K11-linked ubiquitination, and proteasome-mediated ubiquitin-dependent protein catabolic process (Figure 8A). The top 10 projects with the highest enrichment within the CC group were anaphase-promoting complex, cytosol, condensed chromosome kinetochore, kinetochore, nucleoplasm, spindle, midbody, centromeric region chromosome, centrosome, and nucleus. In the MF group, the differentially expressed CDCAs and relevant genes appeared to be chiefly enriched in protein binding and phosphatase binding, histone kinase activity, protein kinase activity, protein serine/threonine kinase activity, anaphase-promoting complex binding, cyclin-dependent protein serine/threonine kinase activity, ATP binding, and microtubule motor activity. The top 10 KEGG pathways significantly related to the tumorigenesis and progression of STAD were the cell cycle, oocyte meiosis, progesterone-mediated oocyte maturation, ubiquitin-mediated proteolysis, human T-lymphotropic virus type-1infection, foxO signaling pathway, vital carcinogenesis, p53 signaling pathway, small cell lung carcinoma, Epstein–Barr virus infection, and hepatitis B (Figure 8B).

### Infiltration analysis of immune cells in relation to CDCAs in patients with STAD

**FIGURE 9:**
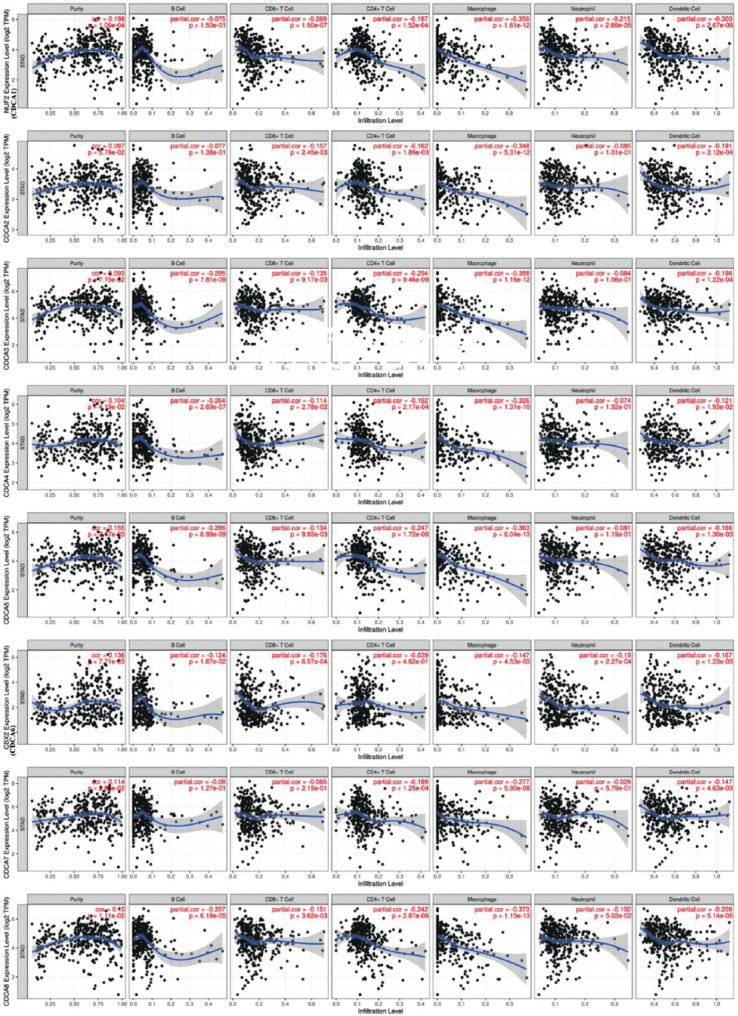
Connections between different cell division cycle-associated proteins (CDCAs) and the infiltrating extents of immunocytes (TIMER). CDCA levels were correlated with the extent of immunological infiltration of immunocytes (*p* < 0.05).

Immunocyte levels are linked with the growth and progression of carcinoma cells. Therefore, using the TIMER database, we assessed the correlation between CDCAs and immunological infiltration (Figure 9). CDCA1 (NUF2) expression was negatively correlated to the immunological infiltration of CD8^+^ T cells (Cor = −0.269, *p* = 1.50E−7), CD4^+^ T cells (Cor = −0.197, *p* = 1.52E−4), macrophages (Cor = −0.356, *p* = 1.61E−12), neutrophils (Cor = −0.215, *p* = 2.86E−5), and dendritic cells (Cor = −0.303, *p* = 2.67E−9). A reverse trend was observed between CDCA2 expression and the infiltration of CD8^+^ T cells (Cor = −0.157, *p* = 2.45E−3), CD4^+^ T cells (Cor = −0.162, *p* = 1.89E−3), macrophages (Cor = −0.348, *p* = 5.31E−12), and dendritic cells (Cor = −0.191, *p* = 2.12E−4). CDCA3 expression was negatively correlated to the infiltration of B cells (Cor = −0.295, *p* = 7.81E−9), CD8^+^ T cells (Cor = −0.135, *p* = 9.17E−3), CD4^+^ T cells (Cor = −0.294, *p* = 9.46E−9), macrophages (Cor = −0.358, *p* = 1.16E−12), and dendritic cells (Cor = −0.198, *p* = 1.22E−4). Similarly, CDCA4 expression was negatively correlated to the infiltration of B cells (Cor = −0.264, *p* = 2.69E−7), CD8^+^ T cells (Cor = −0.114, *p* = 2.78E−2), CD4^+^ T cells (Cor = −0.192, *p* = 2.17E−4), macrophages (Cor = −0.326, *p* = 1.31E−10), and dendritic cells (Cor = −0.121, *p* = 1.93E−2). CDCA5 expression was negatively correlated to the infiltration of B cells (Cor = −0.296, *p* = 6.98E−9), CD8^+^ T cells (Cor = −0.134, *p* = 9.93E−3), CD4^+^ T cells (Cor = −0.247, *p* = 1.72E−6), macrophages (Cor = −0.363, *p* = 6.04E−13), and dendritic cells (Cor = −0.166, *p* = 1.30E−3). CDCA6 (CBX2) expression was negatively correlated to the infiltration of B cells (Cor = −0.124, *p* = 1.67E−2), CD8^+^ T cells (Cor = −0.176, *p* = 6.57E−4), macrophages (Cor = −0.147, *p* = 4.53E−3), neutrophils (Cor = −0.19, *p* = 2.27E−4), and dendritic cells (Cor = −0.167, *p* = 1.23E−3). CDCA7 expression was negatively correlated to the infiltration of CD4^+^ T cells (Cor = −0.199, *p* = 1.25E−4), macrophages (Cor = −0.277, *p* = 5.90E−8), and dendritic cells (Cor = −0.147, *p* = 4.63E−3). CDCA8 expression was negatively correlated to the infiltration of B cells (Cor = −0.207, *p* = 6.18E−5), CD8^+^ T cells (Cor = −0.151, *p* = 3.62E−3), CD4^+^ T cells (Cor = −0.242, *p* = 2.87E−6), macrophages (Cor = −0.373, *p* = 1.15E−13), and dendritic cells (Cor = −0.209, *p* = 5.14E−5).

## DISCUSSION

Currently, the mortality rates of GC remain high, with the number of deaths accounting for 8.2% of all deaths related to different cancer types [1]. Such poor outcomes of GC prompt the search for appropriate prognostic biomarkers.

Consisting of CDCA1–8, the CDCA protein family plays an essential role in cell division. Studies have reported that members of the CDCA family are involved in carcinoma proliferation, apoptosis, invasion, and medication tolerance [34-36]. CDCAs also participate in many pathways related to cancers. For example, CDCA2 modulates cyclin D1 expression as a result of PI3K/AKT pathway activation; thus, promoting the development of colorectal carcinoma cells [37]. CDCA5 disrupts the cellular behavior of liver cell tumors through the AKT pathway [38]. CDCA6 (CBX2) was reported to be strongly associated with the Hippo pathway and yes-associated protein in liver cancer cells [39]. However, the distinctive functions of CDCAs in STAD must be further explored. Here, the prognostic value and biofunctions of CDCAs in STAD were comprehensively analyzed.

Initially, we explored the transcriptional expression profiles of CDCAs and their correlation with the pathological phases of STAD. All eight genes were significantly upregulated in STAD compared with normal tissue. Moreover, patients with STAD and high CDCA4, CDCA7, and CDCA8 expression levels were notably linked with shorter OS. Furthermore, increased levels of CDCA7 in patients with STAD were correlated with poorer DFS. CDCA4 can modulate proliferation and apoptosis in carcinomas by differentially taking control of the transcriptional activity of E2Fs and p53 [14,40]. Studies have demonstrated that CDCA7 knockdown limits the migration of cancer cells by regulating tubulin and actomyosin cytoskeleton dynamics in lymphoma [35]. CDCA8 knockdown is reported to inhibit Rho-associated kinase signaling, inhibiting the proliferation and invasion of cutaneous melanoma cells [41]. These findings led to the hypothesis that CDCAs, especially CDCA4, CDCA7, and CDCA8, may have a substantial impact on STAD.

Besides the various genomic expression in STAD, genomic mutations together with epigenetic changes affect tumor progression [42]. Therefore, the molecular characteristics of CDCAs in STAD were explored in this study. Certain gene changes in CDCAs in STAD were noticed, including decreased mRNA levels, suggesting underlying roles of CDCAs in STAD. Alterations in protein functions such as the regulation of proteins by upstream stimulatory factor 2 might influence cancer progression [43]. Mutations in CDCA1, CDCA3, CDCA4, and CDCA8 were shown to impair protein functions, which might be adverse for the prognosis of patients with STAD.

The PPI network from STRING and GeneMANIA demonstrated that CDCAs are closely linked with the cell cycle, mitosis, and nuclear division. Drugs that regulate the cell cycle might help treat cancers such as breast cancer [44]. Mitosis was reported to be a critical period, during which cells under surveillance might represent targets to inhibit tumor growth [45]. Abnormal nuclear division caused by chromosome instability when the biogenesis factor NOP53 was reduced was involved in pathologies and cancers [46].

Next, we performed functional enrichment analysis. The functional characteristics of the genes were closely associated with the cell cycle, foxO signaling pathway, and Epstein–Barr virus infection. Some studies have shown that the cell cycle is intimately involved in tumorigenesis and cancer progression and strongly influences proliferation and senescence [47-51]. FoxO belongs to the transcription factor family forkhead box and is involved in cellular differentiation, cellular proliferation, apoptosis, and DNA injury and repair and has been a therapeutic target in cancer [52]. Epstein–Barr virus-associated gastric malignancy has become a subtype, which reflects the critical role of this virus in STAD [53].

Increasing evidence suggests that immunocyte infiltration is a crucial pathway influencing tumor progression and relapse [54]. Here, CDCA levels were correlated to the immunological infiltrating extents of several immunocytes, including B cells, CD8^+^ T cells, CD4^+^ T cells, macrophages, neutrophils, and dendritic cells. This indicates that CDCAs may also reflect the immunological conditions besides the prognosis of the tumor. The reactivity of CD8^+^ T cells that is enhanced by inhibiting CD155 and TIGIT (a T cell surface molecule) might improve the survival of mice with GC [55]. These antitumor effects were also observed in CD4^+^ T cells and macrophages: the polarization of M1 macrophages activated CD4^+^ T cells, facilitating cancer cell elimination in lung carcinoma [56]. B cells can exert antitumor effects through different pathways and initiating humoral immunity [57]. The dendritic cell vaccine showed great promise in cancers such as lung cancer [58]. CDCAs were shown to obviously reduce these immune cells in STAD, possibly representing the cause of poor patient prognosis. These results can contribute to the development of new immunotherapies. Checkpoint inhibitors have an effect on GC [59], and as CDCAs participate in the cell cycle, blocking them may be effective in improving the prognosis of patients with STAD.

There are several limitations to our research. All the data analyzed were derived from different online databases, potentially causing background heterogeneity. Further cellular studies along with clinical research are necessary to confirm our results and investigate the underlying mechanisms of the possible roles of CDCAs in STAD.

## CONCLUSION

This study suggests that differentially expressed CDCAs may represent potential biomarkers for the prognosis of patients with STAD and may offer new insight for designing innovative immunotherapies.
